# Normality Tests for Statistical Analysis: A Guide for Non-Statisticians

**DOI:** 10.5812/ijem.3505

**Published:** 2012-04-20

**Authors:** Asghar Ghasemi, Saleh Zahediasl

**Affiliations:** 1 Endocrine Research Center, Research Institute for Endocrine Sciences, Shahid Beheshti University of Medical Sciences, Tehran, IR Iran

**Keywords:** Normality, Statistical Analysis

## Abstract

Statistical errors are common in scientific literature and about 50% of the published articles have at least one error. The assumption of normality needs to be checked for many statistical procedures, namely parametric tests, because their validity depends on it. The aim of this commentary is to overview checking for normality in statistical analysis using SPSS.

## 1. Background

Statistical errors are common in scientific literature, and about 50% of the published articles have at least one error (1). Many of the statistical procedures including correlation, regression, t tests, and analysis of variance, namely parametric tests, are based on the assumption that the data follows a normal distribution or a Gaussian distribution (after Johann Karl Gauss, 1777–1855); that is, it is assumed that the populations from which the samples are taken are normally distributed (2-5). The assumption of normality is especially critical when constructing reference intervals for variables (6). Normality and other assumptions should be taken seriously, for when these assumptions do not hold, it is impossible to draw accurate and reliable conclusions about reality (2, 7).

With large enough sample sizes (> 30 or 40), the violation of the normality assumption should not cause major problems (4); this implies that we can use parametric procedures even when the data are not normally distributed (8). If we have samples consisting of hundreds of observations, we can ignore the distribution of the data (3). According to the central limit theorem, (a) if the sample data are approximately normal then the sampling distribution too will be normal; (b) in large samples (> 30 or 40), the sampling distribution tends to be normal, regardless of the shape of the data (2, 8); and (c) means of random samples from any distribution will themselves have normal distribution (3). Although true normality is considered to be a myth (8), we can look for normality visually by using normal plots (2, 3) or by significance tests, that is, comparing the sample distribution to a normal one (2, 3). It is important to ascertain whether data show a serious deviation from normality (8). The purpose of this report is to overview the procedures for checking normality in statistical analysis using SPSS.

## 2. Visual Methods

Visual inspection of the distribution may be used for assessing normality, although this approach is usually unreliable and does not guarantee that the distribution is normal (2, 3, 7). However, when data are presented visually, readers of an article can judge the distribution assumption by themselves (9). The frequency distribution (histogram), stem-and-leaf plot, boxplot, P-P plot (probability-probability plot), and Q-Q plot (quantile-quantile plot) are used for checking normality visually (2). The frequency distribution that plots the observed values against their frequency, provides both a visual judgment about whether the distribution is bell shaped and insights about gaps in the data and outliers outlying values (10). The stem-and-leaf plot is a method similar to the histogram, although it retains information about the actual data values (8). The P-P plot plots the cumulative probability of a variable against the cumulative probability of a particular distribution (e.g., normal distribution). After data are ranked and sorted, the corresponding z-score is calculated for each rank as follows: *z = x - ᵪ̅ / s*. This is the expected value that the score should have in a normal distribution. The scores are then themselves converted to z-scores. The actual z-scores are plotted against the expected z-scores. If the data are normally distributed, the result would be a straight diagonal line (2). A Q-Q plot is very similar to the P-P plot except that it plots the quantiles (values that split a data set into equal portions) of the data set instead of every individual score in the data. Moreover, the Q-Q plots are easier to interpret in case of large sample sizes (2). The boxplot shows the median as a horizontal line inside the box and the interquartile range (range between the 25 ^th^ to 75 ^th^ percentiles) as the length of the box. The whiskers (line extending from the top and bottom of the box) represent the minimum and maximum values when they are within 1.5 times the interquartile range from either end of the box (10). Scores greater than 1.5 times the interquartile range are out of the boxplot and are considered as outliers, and those greater than 3 times the interquartile range are extreme outliers. A boxplot that is symmetric with the median line at approximately the center of the box and with symmetric whiskers that are slightly longer than the subsections of the center box suggests that the data may have come from a normal distribution (8).

## 3. Normality Tests

The normality tests are supplementary to the graphical assessment of normality (8). The main tests for the assessment of normality are Kolmogorov-Smirnov (K-S) test (7), Lilliefors corrected K-S test (7, 10), Shapiro-Wilk test (7, 10), Anderson-Darling test (7), Cramer-von Mises test (7), D’Agostino skewness test (7), Anscombe-Glynn kurtosis test (7), D’Agostino-Pearson omnibus test (7), and the Jarque-Bera test (7). Among these, K-S is a much used test (11) and the K-S and Shapiro-Wilk tests can be conducted in the SPSS Explore procedure (Analyze → Descriptive Statistics → Explore → Plots → Normality plots with tests) (8).

The tests mentioned above compare the scores in the sample to a normally distributed set of scores with the same mean and standard deviation; the null hypothesis is that “sample distribution is normal.” If the test is significant, the distribution is non-normal. For small sample sizes, normality tests have little power to reject the null hypothesis and therefore small samples most often pass normality tests (7). For large sample sizes, significant results would be derived even in the case of a small deviation from normality (2, 7), although this small deviation will not affect the results of a parametric test (7). The K-S test is an empirical distribution function (EDF) in which the theoretical cumulative distribution function of the test distribution is contrasted with the EDF of the data (7). A limitation of the K-S test is its high sensitivity to extreme values; the Lilliefors correction renders this test less conservative (10). It has been reported that the K-S test has low power and it should not be seriously considered for testing normality (11). Moreover, it is not recommended when parameters are estimated from the data, regardless of sample size (12).

The Shapiro-Wilk test is based on the correlation between the data and the corresponding normal scores (10) and provides better power than the K-S test even after the Lilliefors correction (12). Power is the most frequent measure of the value of a test for normality—the ability to detect whether a sample comes from a non-normal distribution (11). Some researchers recommend the Shapiro-Wilk test as the best choice for testing the normality of data (11).

## 4. Testing Normality Using SPSS

We consider two examples from previously published data: serum magnesium levels in 12–16 year old girls (with normal distribution, n = 30) (13) and serum thyroid stimulating hormone (TSH) levels in adult control subjects (with non-normal distribution, n = 24) (14). SPSS provides the K-S (with Lilliefors correction) and the Shapiro-Wilk normality tests and recommends these tests only for a sample size of less than 50 (8).

In Figure, both frequency distributions and P-P plots show that serum magnesium data follow a normal distribution while serum TSH levels do not. Results of K-S with Lilliefors correction and Shapiro-Wilk normality tests for serum magnesium and TSH levels are shown in Table. It is clear that for serum magnesium concentrations, both tests have a p-value greater than 0.05, which indicates normal distribution of data, while for serum TSH concentrations, data are not normally distributed as both p values are less than 0.05. Lack of symmetry (skewness) and pointiness (kurtosis) are two main ways in which a distribution can deviate from normal. The values for these parameters should be zero in a normal distribution. These values can be converted to a z-score as follows:

*Z _Skewness_= Skewness-0 / SE _Skewness_* and *Z _Kurtosis_= Kurtosis-0 / SE _Kurtosis_*.

**Figure:**
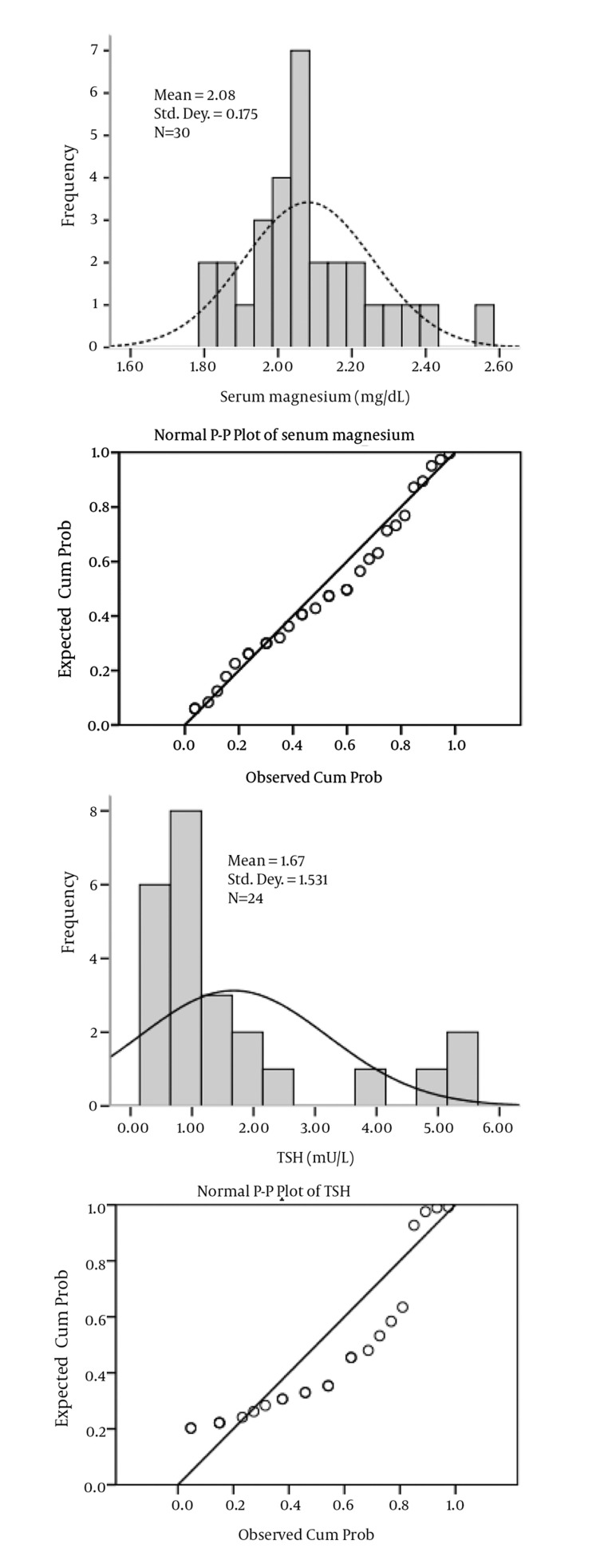
Histograms (Left) and P-P Plots (Right) for Serum Magnesium and TSH Levels

**Table:** Skewness, kurtosis, and Normality Tests for Serum Magnesium and TSH Levels Provided by SPSS

	No.	Mean ± SD a	Mean ± SEM a	Skewness	SE_Skewness_	Z_Skewness_	Kurtosis	SE_Kurtosis_	Z_Kurtosis_	K-S a With Lilliefors Correction Test				Shapiro-Wilk Test		
										Statistics		Df a	*P* value	Statistics	Df a	*P*-value
Serum magnesium, mg/dL	30	2.08 ± 0.175	2.08 ± 0.03	0.745	0.427	1.74	0.567	0.833	0.681	0.137		30	0.156	0.955	30	0.236
Serum TSH a , mU/L	24	1.67 ± 1.53	1.67 ± 0.31	1.594	0.472	3.38	1.401	0.918	1.52	0.230		24	0.002	0.750	24	<0.001

^a^Abbreviations: Df, Degree of freedom; K-S, Kolmogorov-Smirnov; SD, Standard deviation; SEM, Standard error of mean; TSH, Thyroid stimulating hormone

An absolute value of the score greater than 1.96 or lesser than -1.96 is significant at P < 0.05, while greater than 2.58 or lesser than -2.58 is significant at P < 0.01, and greater than 3.29 or lesser than -3.29 is significant at P < 0.001. In small samples, values greater or lesser than 1.96 are sufficient to establish normality of the data. However, in large samples (200 or more) with small standard errors, this criterion should be changed to ± 2.58 and in very large samples no criterion should be applied (that is, significance tests of skewness and kurtosis should not be used) (2). Results presented in Table indicate that parametric statistics should be used for serum magnesium data and non-parametric statistics should be used for serum TSH data.

## 5. Conclusions

According to the available literature, assessing the normality assumption should be taken into account for using parametric statistical tests. It seems that the most popular test for normality, that is, the K-S test, should no longer be used owing to its low power. It is preferable that normality be assessed both visually and through normality tests, of which the Shapiro-Wilk test, provided by the SPSS software, is highly recommended. The normality assumption also needs to be considered for validation of data presented in the literature as it shows whether correct statistical tests have been used.
